# Cuproptosis-based nanomedicine in cancer metastasis synergistic therapy

**DOI:** 10.1016/j.apsb.2025.11.003

**Published:** 2025-11-05

**Authors:** Kan Zhou, Zi-Zhan Li, Yi Liu, Lei-Ming Cao, Han-Yue Luo, Guang-Rui Wang, Kang-Ning Wang, Jinmei Wu, Bing Liu, Zhiyong Song, Lin-Lin Bu

**Affiliations:** a State Key Laboratory of Oral & Maxillofacial Reconstruction and Regeneration, Key Laboratory of Oral Biomedicine Ministry of Education, Hubei Key Laboratory of Stomatology, School & Hospital of Stomatology, Wuhan University, Wuhan 430079, China; b Department of Oral & Maxillofacial-Head Neck Oncology, School & Hospital of Stomatology, Wuhan University, Wuhan 430079, China; c Key Laboratory of Combinatorial Biosynthesis and Drug Discovery, School of Pharmaceutical Sciences, Wuhan University, Wuhan 430071, China; d National Key Laboratory of Agricultural Microbiology, College of Chemistry, Huazhong Agricultural University, Wuhan 430070, China

**Keywords:** Cuproptosis, Cancer metastasis, Nanomedicine, Immunotherapy, Synergistic therapy

## Abstract

Cancer metastasis is a critical indicator of cancer progression and serves as a major cause of cancer-related deaths. Cuproptosis is a novel form of regulated cell death proposed in 2022. Unlike ferroptosis and other known regulated cell deaths (RCDs), cuproptosis has a unique regulatory pathway, and its major biochemical features include copper overload, lipoylated tricarboxylic acid cycle protein aggregation, and the loss of iron-sulfur cluster protein. Cuproptosis-based nanomedicine provides novel therapeutic insights for metastatic cancer treatment. The close link between cuproptosis and cancer therapy has been explored, and several therapeutic strategies have been developed, including copper ionophores and drug delivery systems. Cuproptosis-based nanotherapeutic strategies may enable controlled and selective drug release, and through the multifaceted actions of copper metallocompounds, achieve multimodal theranostic modalities or organically synergize with other regulated RCD pathways. This integration enhances antitumor effects and biosafety, overcomes tumor resistance, and improves the tumor microenvironment. In this review, we systematically delineate the mechanisms of cuproptosis and its current therapeutic implications in metastatic malignancies. We further critically analyze these synergistic therapeutic approaches, prospect emerging applications of cuproptosis-based nanomedicine in metastatic oncology, and highlight their untapped therapeutic potential. Ultimately, we anticipate this exploration will inform innovative clinical management strategies for cancer patients with metastasis.

## Graphical abstract

This review summarizes recent advances in cuproptosis-based nanomedicine for cancer metastasis, focusing on molecular mechanisms, drug delivery strategies, and potential for synergistic therapy, thereby advancing precision treatment for cancer metastasis.

**Figure:**
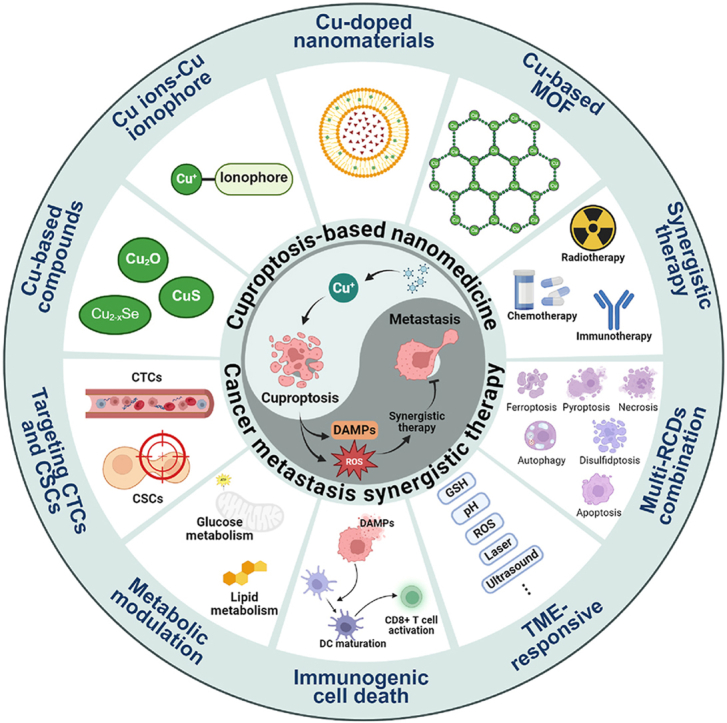

## 1 Introduction

Cancer is a major public health problem worldwide, and the number of cancer patients and deaths from cancer is currently increasing every year^1^^,^^2^. This grim situation has led to a desire for advanced therapeutic approaches. Cancer metastasis, a hallmark of disease progression encompassing lymph node metastasis, distant metastasis, and other dissemination routes, triggers the collapse of systemic antitumor immunity^3^. This pathophysiological cascade underlies cachexia development while contributing significantly to cancer-related mortality. Metastatic cancer extends beyond the realm of surgical intervention, necessitating adjuvant therapies to optimize patient outcomes even when surgery is performed. Multimodal therapeutic strategies encompassing radiotherapy and pharmacotherapy, while improved by current adjunctive approaches and clinical trials in terms of patient survival, may find superior potential applications through novel mechanism-based drug treatments or radiotherapy sensitization mechanisms^4^^,^^5^. Although current multimodal regimens have refined clinical management of metastatic malignancies, the suboptimal overall survival rates persist. This therapeutic impasse demands paradigm-shifting combinatorial therapeutic strategies to achieve meaningful survival prolongation^6^^,^^7^.

**Figure 1:**
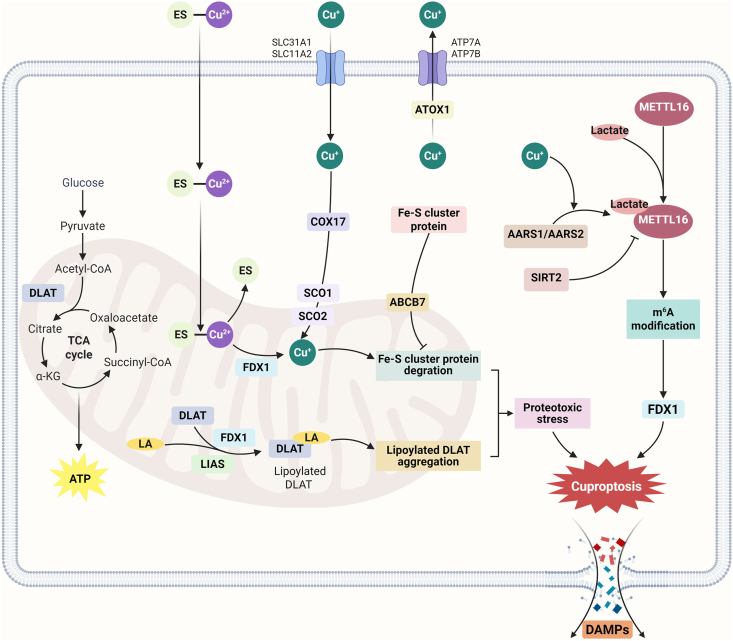
Mechanism of cuproptosis. Copper ionophores can transport Cu^2+^ into cells, recognize ferredoxin 1 (FDX1), and reduce Cu^2+^ to Cu ^+^ under the catalysis of FDX1. Excessive copper ions, on the one hand, directly bind to acylated TCA cycle proteins, causing oligomerization of acylated proteins; On the other hand, it leads to the loss of intracellular iron-sulfur cluster proteins (Fe–S cluster protein). The two will cause cellular protein toxicity stress and lead to cuproptosis. In addition, copper stress promotes lactylation of METTL16, which is a critical regulator of cuproptosis through the m^6^A modification on *FDX1* mRNA. ABCB7 is the transport protein on mitochondria, which transports Fe–S cluster protein into mitochondria and inhibits the cuproptosis. ATOX1, Antioxidant 1 Cu chaperone; ATP7A, ATPase copper transporting *α*; ATP7B, ATPase copper transporting *β*; COX17, cytochrome *c* oxidase copper chaperone; DLAT, dihydrolipoamide *S*-acetyltransferase; ES, elesclomol; LA, lipoic acid; LIAS, lipoyl synthase; SCO1, synthesis of cytochrome *c* oxidase 1; SCO2, synthesis of cytochrome *C* oxidase 2; SLC31A1/CTR1, solute carrier family 31 member 1; SLC11A2/DMT1, solute carrier family 11 member 2; TCA, tricarboxylic acid. Created with BioRender.com.

Cuproptosis, a form of copper (Cu) dependent regulated cell death (RCD), was first described in 2022 by Tsvetkov et al.^8^, who conducted an intensive study of elesclomol (ES, Cu ionophore)-mediated cuproptosis. Unlike other known RCDs (such as apoptosis, necroptosis, pyroptosis, and ferroptosis), cuproptosis is characterized by a unique regulatory pathway associated with Cu ^+^ overload^9^. It's found that excess Cu^+^ in mitochondria directly binds to the lipoylated proteins of the tricarboxylic acid (TCA) cycle, leading to the disulfide-bond-dependent aggregation of these lipoylated proteins. In addition, Cu^+^ can also induce the destabilization of the iron-sulfur cluster proteins (Fe–S cluster proteins), which are involved in many essential processes of mitochondrial function^10^. Subsequently, aggregated lipoylated proteins of the TCA cycle and Fe–S cluster proteins combine to cause proteotoxic stress, ultimately contributing to cuproptosis^11^^,^^12^. Furthermore, some key molecules in cuproptosis were identified by genome-wide knockout, including ferredoxin 1 (FDX1), components of the lipoic acid pathway, and pyruvate dehydrogenase (PDH) complex. In the earlier work of Tsvetkov et al.^8^^,^^13^, FDX1 has proven to be a direct target of ES, and ES-Cu^2+^ can be used as a substrate for FDX1 to produce the more toxic Cu^+^. FDX1 belongs to a family of ferredoxins consisting of Fe–S cluster proteins, and FDX1 functions as an electron transporter. In the lipoylation reaction, FDX1 initiates this lipoyl synthase-dependent biological reaction by particularly providing electrons and target cuproptosis^14^. Dihydrolipoamide *S*-acetyltransferase is one of the four known lipoylases in mitochondria and a subunit of the PDH complex, which binds to and aggregates with Cu^+^-binding lipoic acids in mitochondria^15^. More signaling pathways involved in regulating cuproptosis are still being explored. Sun et al.^16^ found that intracellular excess Cu ^+^ promotes the lactylation of METTL16 and induces cuproptosis through the m^6^A modification on *FDX1* mRNA. Meanwhile, a recent study has identified a new mechanism by which the lack of Fe–S cluster proteins induces cuproptosis. They found that overexpression of the mitochondrial Fe–S cluster protein transporter protein ATP binding cassette subfamily B member 7 would help rescue Cu ionophores-induced cuproptosis, and that downregulation of ATP binding cassette subfamily B member expression by a mutation in *SF3B1* in patients with acute myelogenous leukemia led to a genetic susceptibility to cuproptosis (Fig. 1)^17^.

**Figure 2:**
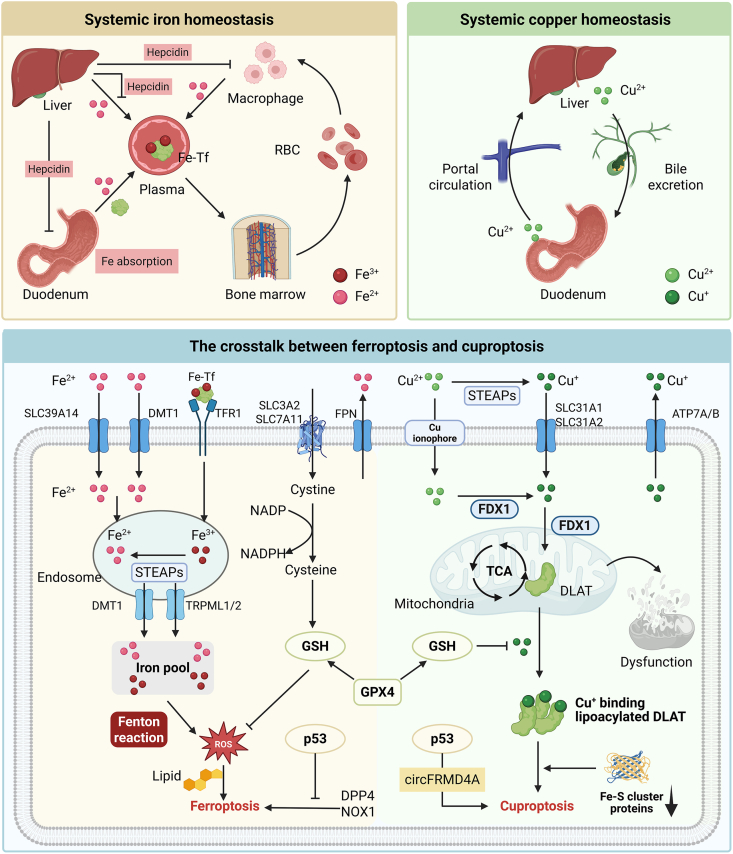
Systemic homeostasis of Fe and Cu in humans and the role of GSH in cuproptosis and ferroptosis. In cuproptosis, glutathione (GSH) prevents the DLAT lipoacylation procedure and FDX1-mediated Cu^+^ binding to lipoacylated DLAT, which prevents cuproptosis. In ferroptosis, GSH plays an important role in maintaining the function of glutathione peroxidase 4 (GPX4), which reduces reactive oxygen (ROS), and GSH is reductive, blocking the oxidation of ROS. DMT1, divalent metal transporter 1. Created with BioRender.com.

The levels of Cu^2+^/Cu^+^ and other transition metal ions are tightly regulated in cells and organisms. Too low a level is detrimental to some biological reactions that depend on metal-binding enzymes, and too high a level can produce metal toxicity^15^. Also, as transition metal element-induced cell death, the crosstalk between ferroptosis and cuproptosis is still partially obscure^18^. There are similar characteristics between iron (Fe) and Cu, both of which are essential elements for serving as enzymatic cofactors and fundamental biological processes, including redox reactions, electron transport, oxygen transport, and energy metabolism^19^^,^^20^. Meanwhile, Fe and Cu are believed to play key roles in promoting a variety of RCDs, including ferroptosis, cuproptosis, apoptosis, necroptosis, autophagy, pyroptosis, etc^19^^,^^21^, ^22^, ^23^. Ferroptosis has gained comprehensive interest for its unique mechanism in tumor suppression, which is characterized by altered Fe homeostasis, oxidative stress, and abnormal lipid peroxidation^24^, ^25^, ^26^. Glutathione peroxidase 4 (GPX4) scavenges lipid peroxidation, the culprit for ferroptosis, in a glutathione (GSH)-dependent manner^27^. It was found that Cu ions bind to specific cysteine residues of the GPX4 protein and promote autophagic degradation of GPX4, which promotes ferroptosis^22^. Meanwhile, the consumption of GSH will also facilitate cuproptosis^18^. Zhang et al.^28^ found that disulfiram (DSF)-Cu can cause both ferroptosis and cuproptosis in Hepatocellular carcinoma cells, and GSH is located at the intersection of the regulatory network. The promotional role of p53 for ferroptosis has long been recognized, and its regulatory mechanism for cuproptosis deserves more in-depth study^29^. Besides promoting reactive oxygen species (ROS) production and inhibiting the expression of solute carrier family 7 member 11 (SLC7A11), p53 may promote cuproptosis by inhibiting glycolysis and driving a metabolic switch towards oxidative phosphorylation^12^. Recent studies have further demonstrated that p53 activation increases the susceptibility of tumor cells to cuproptosis *via* a circular RNA called circFRMD4A. Liao et al.^30^ found that circFRMD4A shifted glucose metabolism in tumor cells from the pyruvate direction to the TCA cycle by inhibiting pyruvate kinase isozyme typeM2. p53 agonists in combination with ES achieved significant tumor suppression in a mouse model. In addition, the connections between ferroptosis and cuproptosis include the following: (1) Ferroptosis depends on glucose uptake and pyruvate oxidation, while cuproptosis depends on mitochondrial respiration. Under hypoxia (1% O_2_) conditions, cells are more dependent on glycolysis and have a reduced sensitivity to cuproptosis^31^^,^^32^. (2) Fe^2+^ is catalytically active in the Fenton reaction, while Cu^2+^ and Cu ^+^ exhibit higher Fenton-like catalytic activity over a wider pH range. (3) Compared to ferroptosis, ROS is not necessary for cuproptosis, as can be seen from the inability of antioxidants such as *N*-acetylcysteine to rescue the growth inhibition caused by ES^31^. (4) Both cuproptosis and ferroptosis can serve as immunogenic cell death (ICD), which is accompanied by exposure of dead cell fragments and damage-associated molecular patterns (DAMPs, including ATP, high mobility histone B1, calcium reticulin, and heat shock protein 90) that can serve as antigens^33^^,^^34^. DAMPs will promote the recognition and presentation of cancer antigens by antigen-presenting cells, improve the tumor microenvironment (TME), and enhance the effectiveness of cancer immunotherapy (Fig. 2).

Crosstalk between cuproptosis and multiple RCDs might be tightly linked to cellular metabolism. For example, cuproptosis is closely linked to glucose metabolism, while ferroptosis is closely linked to lipid metabolism and redox homeostasis. SLC7A11-mediated uptake of cystine suppresses ferroptosis; however, this may lead to another new form of metabolism-related RCD called disulfidptosis^35^. In cancer cells with overexpression of *SLC7A11*, the combination of glucose starvation and high rates of cystine uptake will deplete the NADPH pool, which further results in massive accumulation of disulfides and triggers disulfidptosis^36^. These metabolic RCDs above are closely related to a variety of fundamental metabolisms, and metabolic reprogramming is one of the important hallmarks of malignancy^37^^,^^38^. Compared to non-metastatic primary tumors, metastatic primary tumors harbor subpopulations exhibiting resistance to both ferroptosis and cuproptosis, defined as metal-dependent cell death resistance^39^. These metal-dependent cell death resistance subpopulations demonstrate enhanced glycolytic flux, GSH metabolism, and hypoxic activity while displaying more pronounced epithelial–mesenchymal transition signatures. Such characteristics potentially drive lymph node metastasis in oral carcinomas and show strong associations with resistance to both immunotherapy and EGFR-targeted therapies^40^. Ferroptosis has developed several metabolic pathways for cancer therapy, and newly found cuproptosis and disulfidptosis also have great potential in cancer metabolic therapy. Triggering metabolic RCDs by targeting reprogrammed metabolism can be a promising strategy to effectively and precisely target the vulnerability of tumors, reverse drug resistance, and remodel TME^41^^,^^42^ (Table 1^8^^,^^36^^,^^43^^,^^44^).

**Table 1:** Comparison between cuproptosis, ferroptosis, and disulfidptosis.

Form	Morphological feature	Biochemical feature	Immunological feature	Key regulator	Ref.
Cuproptosis	Mitochondrial volume decreases, membrane density increases, and even swelling and cavitation in mitochondria	Intracellular copper accumulation, direct linkage of copper to lipoylated proteins with disulfide bonds, oligomerization of lipoylated TCA cycle proteins and loss of Fe–S cluster proteins	Pro-inflammatory due to release of TAAs and DAMPs (*e.g.*, ATP, HMGB1, CRT)	Positive • FDX1 • LIAS • LIPT1 • DLD • DLAT • PHDA1 • PDHB • p53 • AARS1/AARS2 • METTL16 Negative • MTF1 • GLS • CDKN2A • ATOX1 • ABCB7 • SIRT2	8,43
Ferroptosis	Characteristic rounded shape resembling necrotic cells Cell membrane: No rupture or blistering of the plasma membrane Cytoplasm: Smaller mitochondria, increased density of mitochondrial membranes, decreased or absent mitochondrial cristae, ruptured outer mitochondrial membranes; no swelling of cytoplasm or organelles. Nucleus: Normal nuclear size and structure, no chromatin condensation	Fe and ROS accumulate MAPKs in the activation of the inhibitory system Xc-, decreasing cystine uptake, GSH depletion, and increasing NADPH oxidation, releasing arachidonic acid mediators	Pro-inflammatory due to release of DAMPs (*e.g.*, ATP, HMGB1, CRT)	Positive • VDAC2/3 • Ras • NOX • TFR1 • p53 • CARS Negative • GPX4 • SLC7A11 • HSPB1 • NRF2	44
Disulfidptosis	a	Aberrant accumulation of intracellular Disulfides, glucose starvation, aberrant disulfide bonds in actin cytoskeleton proteins, F-actin Collapse	a	Positive • SLC7A11 • Rac Negative • WRC	36

a Means unknown content. CRT, calcium reticulin; DAMPs, damage associated molecular patterns; HMGB1, high mobility histone B1; FDX1, ferredoxin 1; LIAS, lipoic acid synthetase; LIPT1, lipoyltransferase 1; DLD, dihydrolipoamide dehydrogenase; DLAT, dihydrolipoamide *S*-acetyltransferase; PDHA1, pyruvate dehydrogenase E1 subunit *α* 1; PDHB, pyruvate dehydrogenase E1 subunit *β*; AARS1, alanyl-tRNA synthetase 1; AARS2, alanyl-tRNA synthetase 2; METTL16, methyltransferase-like protein 16; MTF1, metal regulatory transcription factor 1; GLS, glutaminase; CDKN2A, cyclin-dependent kinase inhibitor 2A; ATOX1, antioxidant 1 copper chaperone; ABCB7, ATP-binding cassette sub-family B member 7; SIRT2, sirtuin 2; VDAC2, voltage-dependent anion channel 2; VDAC3, voltage-dependent anion channel 3; Ras, rat sarcoma viral oncogene homolog; NOX, NADPH oxidase; TFR1, transferrin receptor 1; CARS, cysteinyl-tRNA synthetase; GPX4, glutathione peroxidase 4; SLC7A11, solute carrier family 7 member 11; HSPB1, heat shock protein beta-1; NRF2, nuclear factor erythroid 2–related factor 2; SLC7A11, solute carrier family 7 member 11; Rac, Ras-related C3 botulinum toxin substrate; WRC, WAVE regulatory complex.

Cuproptosis has emerged as a transformative frontier in oncology therapeutics, with seminal studies demonstrating upregulated expression of dihydrolipoamide *S*-acetyltransferase and lipoyl synthase in metastatic lesions compared to primary tumors, suggesting enhanced therapeutic susceptibility of metastatic malignancies to cuproptosis induction^8^. Despite this promise, first-generation Cu ionophores and Cu-based compounds—such as ES, DSF, and NSC319726—exhibit clinically suboptimal tumor response heterogeneity and dose-limiting neurotoxicity, critically impeding their translational utility. Cuproptosis-based nanomedicine modulation addresses these limitations through tumor-targeted drug delivery systems and functionalized nanoparticles (NPs) that achieve spatiotemporally controlled payload release. Such platforms enhance Cu-mediated cytotoxicity while mitigating off-target organotoxicity, with emerging evidence supporting their capacity to override therapeutic resistance *via* Cu-induced metabolic rewiring. The limited enrichment of Cu ions within metastasis restricts the anticancer efficacy of conventional Cu ionophores such as ES when administered *via* standard delivery approaches, representing a key factor contributing to the absence of significant survival benefits in clinical trials. Nanomedicine-based strategies can enhance the delivery of Cu ionophores or Cu-based drugs, particularly through tumor-targeted approaches enabling precise drug delivery or controlled release. Consequently, cuproptosis-based nanomedicine strategies may augment the efficiency of cuproptosis-based cancer therapeutics while potentially achieving superior biosafety profiles to accelerate clinical translation. Current research synergistically integrates cuproptosis activation with other RCDs (*e.g.*, ferroptosis, pyroptosis, apoptosis, and autophagy) and conventional therapies (including phototherapy, immunotherapy, chemotherapy, and radiotherapy), collectively targeting the adaptive mechanisms of metastatic dissemination. This review systematically delineates the molecular circuitry governing cuproptosis and its context-dependent roles in malignant progression and critically analyzes multimodal therapeutic integration strategies. Furthermore, we explore translational prospects of cuproptosis-based nanomedicine in metastasis interception, particularly focusing on underexplored therapeutic dimensions. By bridging mechanistic insights with nanotechnology innovations, this review aims to redefine therapeutic paradigms for metastatic cancers, ultimately striving to deliver clinically meaningful survival benefits to patients with advanced malignancies.

## 2 Current status of cuproptosis in cancer treatment

Prior to the proposal of cuproptosis, the important role of Cu in cancer treatment had already been recognized. The levels of Cu in the serum and tumor tissue of cancer patients, in particular chemotherapy-resistant patients, are significantly higher than those of normal individuals. It has been demonstrated that tumor cells have a high demand for Cu, and Jin et al.^45^ claimed that Cu and its transporting ATPase promote drug resistance *via* their potential mechanism of DNA damage repair. Antioxidant 1 Cu chaperone plays an essential role in Cu transporting, and it also induces the expression of a key protein in DNA damage repair (mediator of DNA damage check point protein 1) by targeting the mediator of DNA damage check point protein 1 promoter. In addition, Cu has proven to be closely related to tumor proliferation, metastasis, and angiogenesis^46^. Cu serves as a cofactor for a host of metalloenzymes that contribute to malignant progression. Moreover, researchers have shown that Cu also regulates cell signaling pathways *via* direct interaction with protein kinases. It has been reported that Cu chelator (tetrathiomolybdate) significantly inhibits cancer metastasis by modulating the adenosine monophosphate-activated protein kinase/mechanistic target of rapamycin complex 1 signaling pathway^47^.

Some powerful Cu ionophores can significantly increase intracellular Cu content, thereby inducing dysregulation of Cu homeostasis and increasing susceptibility to cuproptosis^48^. Before the mechanism of cuproptosis was suggested, some mainstream Cu ionophores have been used as anticancer drugs for cancer treatment. ES has long been used as an anticancer drug targeting mitochondrial metabolism^49^. ES alone or in combination with paclitaxel has completed a series of clinical trials targeting patients with different tumors. There are also reports that DSF can target cancer stem cells (CSCs) in various cancers, and Cu-DSF inhibits acetaldehyde dehydrogenase 1 activity associated with CSCs and enhances the cytotoxicity of anticancer drugs. DSF also shows inhibitory effects in cancer cell proliferation, angiogenesis, drug resistance, and cancer cell metastasis^50^. Furthermore, there is a significant correlation between cuproptosis and the level of mitochondrial metabolism. Some drug-resistant tumors and some CSC-like cells exhibit a higher level of mitochondrial metabolism, which is an attractive target for cancer treatment^51^. There are reports that combining Cu ionophores with targeted therapeutic drugs such as tyrosine kinase inhibitors and protease inhibitors can have better therapeutic effects on tumors with a high mitochondrial metabolic state^52^.

Studies have confirmed that Cu can increase the expression of programmed cell death-ligand 1 (PD-L1). Some studies have been conducted to increase the Cu content by Cu ionophores, thus promoting cuproptosis and increasing the expression of PD-L1 in tumor cells, which is expected to improve the therapeutic effect of *α*PD-L1 (PD-L1 antibody) in immunotherapy^53^. On the other hand, Cu chelators used for reducing plasma Cu levels activate anti-tumor immunity and limit the progression and metastasis of cancer by inhibiting angiogenesis^54^^,^^55^. Furthermore, cuproptosis can also be an ICD by releasing tumor-associated antigens and DAMPs to enhance anti-tumor immunity^56^. Li et al.^57^ successfully induced ICD through a Cu-coordinated covalent organic framework. This attempt demonstrates the great potential of cuproptosis in enhancing immunotherapy. Moreover, the relationship between cuproptosis-related genes (CRGs) and immunotherapy has also received great attention. Zhu et al.^58^ analyzed 1274 colorectal cancer samples based on 16 CRGs and found that three cuproptosis patterns were consistent with three immune infiltration characteristics: immune apoptosis, immune inflammation, and immune rejection. The study revealed a novel cuproptosis-related molecular pattern associated with TME phenotype. Shen et al.^59^ identified four clusters based on CRGs. Observing the relationship between cuproptosis-related clusters and clinical characteristics, prognosis, immune cell infiltration, and chemotherapy sensitivity, a cuproptosis score was constructed. Using cuproptosis score to determine the prognosis of lower-grade glioma patients, it was found that higher scores lead to higher immune infiltration, which is associated with immune checkpoint inhibitors, immune therapy responses, and immune phenotypes. However, the underlying mechanism by which cuproptosis is associated with tumor immunity is still unknown. Further discovery will facilitate the development of strategies to induce cuproptosis to enhance tumor immunotherapy.

The combination of Cu ionophores with anticancer drugs has certain development prospects, but the main drawback of Cu carrier therapy is that Cu delivery does not have targeted properties. The non-selective accumulation of Cu in healthy areas may lead to an increase in toxicity levels. Therefore, targeted delivery methods are demanded to address this issue^60^. Substantial research efforts have been dedicated to developing cuproptosis-based nanocarriers to enhance drug delivery efficiency and optimize biosafety profiles in oncological applications. As summarized in Table 2^43^^,^^53^^,^^61^, ^62^, ^63^, ^64^, ^65^, ^66^, ^67^, ^68^, ^69^, ^70^, ^71^, ^72^, ^73^, ^74^, ^75^, ^76^, ^77^, ^78^, ^79^, ^80^, contemporary cuproptosis-based nanomedicine strategies for cancer therapeutics predominantly focus on primary tumor eradication, while exploration of their efficacy against metastatic lesions remains conspicuously limited.

**Table 2:** Current status of cuproptosis-based nanomedicine for cancer treatment.

Name	Cancer type	Forms of Cu	Treatment method	Drug delivery strategy	Features	Ref.
HaMOF	Breast cancer	Cu^2+^	Amplification of oxidative pressure	HA; Responding to the slightly aidic TME with high GSH	Synergizing with ferroptosis and apoptosis	43
NP@ES-Cu	Bladder cancer	ES-Cu	Immunotherapy	ROS-sensitive	Decreasing immunosuppression and increasing mature DCs and CD8^+^ T cells in TME	53
Au@MSN-Cu/PEG/DSF	Breast cancer	CuET	PTT	EPR effect	High inhibition rate and minimal side effects	61
DMMA@Cu_2-x_Se	Malignant melanoma and cervical cancer	Cu_2-x_Se	PTT	RGD polypeptide; TME-responsive	High efficiency and promotes cuproptosis	62
AuPt@Cu-PDA	Breast cancer	Cu-PDA	PTT/NCT	EPR effect	Multi-enzyme activities and PAI/PTI multi-model imaging	63
GOx@[Cu(tz)]	Breast cancer and bladder cancer	[Cu(tz)]CP	PDT, starvation therapy	GSH-responsive	Suppressing tumor growth with high efficiency and safety	64
NCTD gel	Liver cancer	Cu^2+^	PDT and CDT	Responsive to acidic and H_2_O_2_-overexpressed TME	Anti-inflammatory effects; high specificity and continuous drug release	65
BSO-CAT@MOF-199 @DDM (BCMD)	Glioblastoma	Cu-MOF	Immunotherapy	Responding to the slightly acidic environment of the tumor	Supplying O_2,_ inducing systemic immune response and effectively reversing the immunosuppression of TME	66
^Cu/AP^H-M	Low rectal tumor	Cu^2+^	Immunotherapy and radiotherapy	CT26 cancer cell membrane	Inhibition of distal tumors; immune memory effect; CAT activity	67
CS/MTO-Cu@AMI	Breast cancer	Cu-MTO coordination	Immunotherapy and chemotherapy	CS; pH/GSH-dual responsive	Reversing drug resistance; activating Cgas–STING pathway; inhibition of tumor recurrence and metastasis	68
CuX-P	Triple negative breast cancer	DSF/Cu^2+^	Immunotherapy, PTT	PD-1 overexpressing T cell membrane	Excellent and well-designed mechanism of endocytosis and targeting	69
CCNAs	Prostate cancer	Cu^2+^-coordination	Immunotherapy and PDT	ROS-responsive	IDO inhibitor for TME remodeling	70
CQG NPs	Breast cancer	Cu-quinone	Immunotherapy, pyroptosis, cuproptosis, and starvation therapy	EPR effect	Multi-enzyme activities; combination of cuproptosis and pyroptosis for TME remodeling	71
CuET-BSA	Lung cancer	CuET	Chemotherapy	BSA	Resistant to GSH and tackling CDDP-based drug resistance; low systemic toxicity	72
CSTD-Cu(II)@DSF	Breast cancer	Cu^2+^, DSF	CDT and chemotherapy	Responsive to the slightly acidic TME with high ROS	Integration of diagnosis and treatment	73
Cu-GA NPs	Breast cancer and colon cancer	Cu^+^	CDT and chemotherapy	GSH-responsive	Excellent biosafety	74
HD/BER/GOx/Cu	Breast cancer	Cupric sulfate	CDT and starvation therapy	HA-dopamine-based gel	Low systemic toxicity	75
ZIF-8-Cu_2_O-DNA	Pancreatic cancer	Cu_2_O	CDT	Responsive to acidic TME	Utilizing a DNA enzyme to enhance treatment	76
Au_25_(NAMB)_18_NCs Cu^2+^@SA/HA NHG	Liver cancer	Cu^2+^	PDT, PTT, and CDT	SA/HA hydrogel shell; Responding to the slightly acidic TME with high ROS	Multifactorial triggered TME; MCT; integration of diagnosis and treatment	77
ART@CuT/ETH HNP	Breast cancer	Cu^2+^	Amplification of oxidative pressure	HA; TME-responsive	Synergizing with ferroptosis and apoptosis	78
SonoCu	Breast cancer	Cu^2+^	SDT	MCM; pH-responsive and ultrasound-responsive	Supplying O_2_; high selectivity and permeability	79
HFn-Cu-REGO NP	Glioblastoma	Cu^2+^	Targeted therapy and manipulating autophagy	TfR1-mediated active targeting; pH-responsive	Negligible side effects and excellent suppressive effects	80

ART, artemisinin; BER, berberine; BSA, bovine serum albumin; BSO, buthionine-sulfoximine; CAT, catalase; CDDP, cisplatin (also known as *cis*-diaminodichloroplatinum); CDT, chemodynamic therapy; CP, coordination polymer; CS, chondroitin sulfate; CSCs, cancer stem cells; CSG, ES-Cu/Galactose-Alginate; CSTD, core–shell tecto dendritic polymer; CuET, bis(diethyldithiocarbamate)copper; CuMS, copper loaded metallic molybdenum bisulfide nanosheets; [Cu(tz)], copper(I) 1,2,4-triazolate; DCs, dendric cells; DDM, Dodecyl-*β*-D-maltoside; DMMA, dimethyl maleic anhydride; DSF, disulfiram; EPR, enhanced permeability and retention; ES, elesclomol; GA, gallic acid; GOx, glucose oxidase; GSH, glutathione; HA, hyaluronic acid; HCN, heterogeneous carbon nitride nanosheets; HD, hyaluronic acid-dopamine; HFn, heavy chain ferritin; HNP, hollow nanoplatform; MCM, macrophage cell membrane; MCT, multimodal combined therapy; MOF, metal organic framework; MSN, mesoporous silica-coated gold nanorods; NAMB, *N*-acetyl-l-cysteine and 4-mercaptobenzoic acid; NCs, nanoclusters; NCT, nanocatalytic therapy; NHG, nanohybrid gels; NP, nanoparticle; PDA, polydopamine; PD-L1, programmed cell death 1 ligand 1; PDT, photodynamic therapy; PEG, polyethylene glycol; pH, potential of hydrogen; PTI, photothermal imaging; PTT, photothermal therapy; REGO, regorafenib; RGD, Arg-Gly-Asp; ROS, reactive oxygen; SA, sodium alginate; SDT, sonodynamic therapy; TfR1, transferrin receptor 1; TME, tumor microenvironment.

## 3 Application of cuproptosis-based nanomedicine for cancer metastasis

To address these limitations and maximize the therapeutic utility of cuproptosis, recent research has shifted focus toward integrating targeted delivery systems with combinational treatment strategies. These approaches aim not only to enhance the specificity of Cu accumulation in tumor tissues but also to potentiate the treatment of cancer metastasis through synergistic mechanisms.

**Figure 3:**
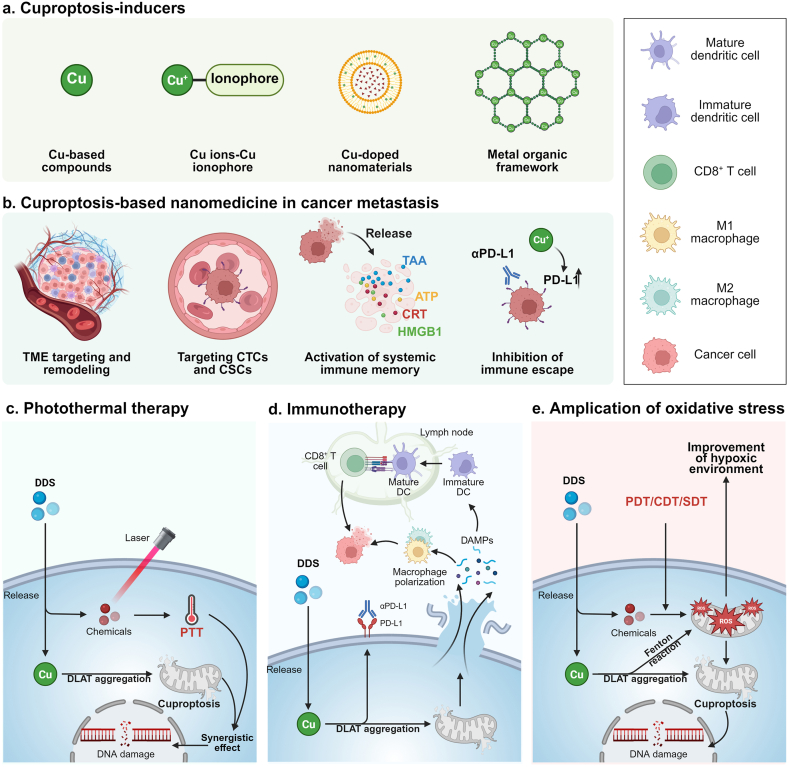
Cuproptosis-based drug delivery systems (DDSs) in cancer metastasis. Cuproptosis can be induced to occur by various forms of Cu, including Cu compounds, Cu ion complexes with Cu ionophore, Cu-doped nanomaterials, and Cu-based metal organic frameworks. Cuproptosis-based nanomedicines constructed on the basis of these materials have potential in the treatment of cancer metastasis. The mechanisms mainly include targeting the tumor microenvironment (TME), thereby reducing recurrence, metastasis, and eliminating cancer circulating cells (CTCs) and tumor stem cells (CSCs). And cuproptosis itself, as immunogenic death (ICD), can induce systemic anti-tumor immune memory. At the same time, Cu can increase the expression of programmed death-ligand 1 (PD-L1) in cancer cells, with immunotherapy, thereby increasing the immune response rate and inhibiting the immune escape of cancer cells. Cuproptosis can, in turn, be combined with other therapies to enhance its anti-metastatic effects, such as photothermal therapy (PTT), immunotherapy, amplification of oxidative stress through photodynamic therapy (PDT)/sonodynamic therapy (SDT)/chemodynamic therapy (CDT), and synergistically with chemotherapy and radiotherapy. CRT, calcium reticulin; DC, dendritic cell; HMGB1, high mobility histone B1; HSP90, heat shock protein 90; TAA, tumor-associated antigens. Created with BioRender.com.

The accumulation of Cu ions in tumor cells is the most important step in inducing cuproptosis in tumor cells. Although the concentration of Cu ions in many tumor tissues is higher than that in normal tissues, the concentration of Cu ions in tumor tissues cannot reach the required concentration to induce cuproptosis in cancer cells. Intracellular Cu homeostasis is regulated, and excess Cu can be excreted from the cell *via* the Cu exporter ATPase copper transporting *β*^8^. In addition, Cu, as a heavy metal ion, has damage to multiple human organs, so strategies to induce cuproptosis should have considerable targeting to reduce treatment side effects. To ensure the good biocompatibility and high selectivity of drugs that induce cuproptosis, and to prevent drug off-target, various new DDSs have been constructed for targeted induction of cuproptosis in tumor cells. More importantly, the therapeutic significance of simply inducing cuproptosis is very limited, and it is necessary to enhance the therapeutic effect by combining it with other treatment methods. Cu-based materials in cuproptosis-based nanomedicine (such as Cu ions, Cu-based oxygenates, Cu-doped nanomaterials, and Cu ligand coordination complexes) can respond to TME-related features (such as acidic microenvironment, high-level ROS, and GSH, higher hypoxic status, etc.) or tumor localization therapy (such as laser and sound stimulation), enabling DDS to deliver Cu to cells in a biodegradable manner (Fig. 3). Further, Cu ions, through their strong Fenton catalytic activity, ability to consume GSH, amplify oxidative stress in tumor cells, and enhance the anti-tumor effects of phototherapy, chemotherapy, and chemodynamic therapy (CDT)^81^.

### 3.1 Combined with phototherapy

Phototherapy, including photodynamic therapy (PDT) and photothermal therapy (PTT), employs near-infrared (NIR) light to target tumor lesions. PDT utilizes photosensitizers in tumor cells to produce single-linear oxygen in response to light irradiation and exert a killing effect^82^. Combining phototherapy with cuproptosis-based DDS enhances antitumor effects, selectivity, and controllable drug release^83^. Ning et al.^84^ developed PTC, a system composed of platelet vesicles, the photosensitizer TBP-2, and Cu_2_O. Platelet vesicles and Cu_2_O provide targeted delivery of TBP-2, and PTC rapidly degrades within acidic tumor cells, releasing Cu^+^ and hydrogen peroxide to trigger cuproptosis and PDT. PTC demonstrated significant therapeutic efficacy in a breast cancer pulmonary metastasis model. Strikingly, the PTC + laser group achieved near-complete suppression of pulmonary metastatic lesions, mechanistically linked to its dual targeting of circulating tumor cells (CTCs) and CSCs through spatiotemporal-controlled eradication. Chen et al.^6^ engineered a hypoxia-modulating nanoplatform (CuET@PHF) through polydopamine and hydroxyethyl starch-stabilized crystallization of the Cu ionophore bis(diethyldithiocarbamate)copper (CuET). This nanosystem harnesses high photothermal conversion efficiency to alleviate tumor hypoxia while simultaneously inducing cuproptosis and eradicating CSCs, achieving dual suppression of tumor recurrence and metastatic dissemination. The combinatorial regimen of CuET@PHF with ionizing radiation demonstrated complete inhibition (100%) of pulmonary metastatic lesions in syngeneic 4T1 models, coupled with durable tumor-specific immunologic memory, evidenced by elevated central memory T cell populations^6^. Ruan et al. designed and prepared a microbial nanohybrid-based DDS (*E. coli*@Cu_2_O) for colon tumor treatment. They utilized the colonizing capacity of *Escherichia coli* to achieve targeted delivery of DDS. Cu_2_O NPs are employed for H_2_S-responsiveness and GSH oxidation, which leads to abundant ROS production and GPX4 inactivation thereby causing cellular ferroptosis and promoting cuproptosis. Moreover, NIR-II (1064 nm) PTT was found to further elevate the therapeutic effects of ferroptosis and cuproptosis. In addition, Ruan et al.^85^ established a classic bilateral injection metastatic model to validate the potent antimetastatic efficacy of the *E. coli*@Cu_2_O and *α*PD-1 combinatorial regimen in colorectal cancer. This engineered therapeutic platform demonstrated systemic eradication of metastatic lesions.

Li et al.^64^ developed a multifunctional Cu-based silk fibroin nanoplatform—denoted as CuS-PEI-siRNA-SFNs — through a layer-by-layer self-assembly strategy. This system integrates a Cu_2-x_S NP core co-loaded with siRNA targeting PD-L1-mediated immune evasion pathways and enveloped within an SF shell. The CuS-PEI-siRNA-SFNs exert antitumor effects by synergistically inducing cuproptosis through PTT, CDT, and immune activation mechanisms. In 4T1 tumor-bearing murine models, the nanoplatform demonstrated preferential accumulation within tumor tissues, effectively suppressing both primary tumor progression and pulmonary metastasis. Notably, this study transcends the simplistic combination of PTT with cuproptosis induction by orchestrating multimodal therapeutic integration, thereby demonstrating enhanced therapeutic benefits^86^.

### 3.2 Combined with immunotherapy

The DDS combines cuproptosis with immunotherapy to enable targeted, controlled, and effective cancer treatments by utilizing the unique properties of Cu along with other therapeutic agents. Combining cuproptosis with radiotherapy and immunotherapy has emerged as a promising approach to cancer treatment, offering synergistic benefits that enhance tumor sensitivity to treatment while bolstering the body's immune response. This integrated strategy maximizes therapeutic efficacy and can lead to long-term immune memory against both primary and distant tumors.

**Figure 4:**
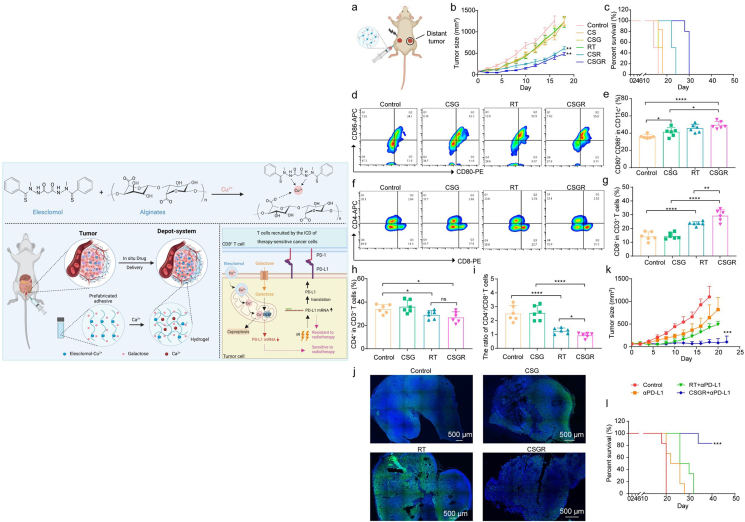
The upper panel demonstrates the preparation of ES-Cu hydrogels and the schematic representation of the inhibition of PD-L1 expression. The lower panel shows a graph of experimental data demonstrating the inhibition of distal tumor growth by ES-Cu-Alginate (CS) and ES-Cu/Galactose-Alginate (CSG) hydrogels enhanced with radiotherapy. The CSGR group (CSG + radiotherapy) showed a significant increase in CD8^+^ T cells at the distal tumor site and a significant decrease in PD-L1. Reprinted with the permission from Ref. 87. Copyright © 2023 American Chemical Society.

Shen et al. designed a hydrogel DDS for cuproptosis-reinforced immuno-radiotherapy, loaded with galactose and ES-Cu^2+^. After local injection, the hydrogel cross-links with Ca^2+^ to form an immobilized DDS in tumors, providing slow and continuous drug release. This local delivery effectively overcomes systemic toxicity and limited solubility of ES-Cu^2+^. The combined therapy significantly downregulates *PD-L1* gene expression, enhancing antitumor immunity and improving the efficacy of immunotherapy and radiotherapy. Moreover, the synergy between cuproptosis and immuno-radiotherapy eradicates primary tumors and triggers a robust immune response that inhibits recurrence (Fig. 4)^87^. Dai et al.^88^ constructed a cuproptosis-based theranostic DDS (PCD@CM), which combines PTT and photoacoustic/fluorescence imaging-dual mode imaging. The DDS consists of self-assembled coordination compounds of Cu^2+^, NIR-II ultrasmall polymer dots, and doxorubicin, and its surface is modified with tumor cell membranes (4T1 cell membrane). The synergistic combination of PCD@CM and *α*PD-L1 significantly attenuated pulmonary metastatic burden in mouse models. Compared to PBS control and *α*PD-L1 monotherapy cohorts, the PCD@CM + *α*PD-L1 combination cohort demonstrated near-complete absence of metastatic foci in lung parenchyma, suggesting potent eradication of disseminated tumor cells. Emerging studies have developed a Cu-erastin co-delivery nanoplatform (CuP/Er) to synergistically induce cuproptosis and ferroptosis in malignant cells. Mechanistically, Erastin sensitizes tumor cells to Cu-mediated cuproptosis by suppressing the Warburg effect—a metabolic reprogramming phenomenon that confers therapeutic resistance. This dual RCD activation strategy demonstrated potent antimetastatic efficacy in both orthotopic colorectal cancer and breast cancer pulmonary metastasis models. Notably, the combinatorial regimen with immune checkpoint blockade therapy elicited durable antitumor immunity characterized by elevated tumor-infiltrating CD8^+^ T cells and memory T cell populations, effectively suppressing established metastasis and preventing tumor recurrence^89^. Yan et al.^90^ pioneered an inhalable poly(2-(*N*-oxide-*N*, *N*-diethylamino)ethyl methacrylate)-coated Cu-based metal organic framework (Cu-MOF) to address the limitations of intravenous delivery in pulmonary metastasis targeting. Synergistic TME reprogramming with *α*PD-L1 achieves complete response rates and prolonged survival in lung metastasis models. This inhalation-driven strategy establishes a new paradigm for metastasis-targeted immunometabolic therapy.

### 3.3 Amplification of oxidative stress

Tumor cells produce high levels of ROS (O_2_^•−^, ^•^OH, and hydrogen peroxide, etc.) through changes in signal transduction and metabolic pathways. Some endogenous antioxidants (GSH, NADPH) play an important role in maintaining intracellular redox homeostasis. By increasing ROS or inhibiting the antioxidant system through various pathways, the redox balance within tumor cells is disrupted. This state is called oxidative stress, which can cause DNA damage to tumor cells and activate p53, thereby slowing down tumor cell proliferation^91^. Liao et al.^7^ engineered X-ray-responsive nanocapsules enabling spatiotemporally controlled Cu ion release, where liberated Cu^+^ ions selectively bind lipoyl-proteins in the TCA cycle to induce cuproptosis *via* Fe–S cluster depletion and lipoylated protein aggregation. This nanoplatform demonstrated dual therapeutic benefits: overcoming breast cancer radioresistance through cuproptosis-driven ICD while suppressing metastatic progression *via* niche disruption. Remarkably, combining the nanocapsules with radiotherapy enhanced CD8^+^ cytotoxic T lymphocyte infiltration in pulmonary metastasis and elicited systemic antimetastatic immunity through enhanced tumor antigen cross-presentation, establishing a paradigm for metastasis-targeted radio-immunotherapy. Lu et al.^92^ developed a celastrol-based Cu nanocomposite (Cel-Cu NP) that leverages the natural product's dual capacity to deplete intracellular GSH and amplify cuproptosis through Cu-mediated proteotoxic stress, thereby disrupting redox homeostasis and overriding GSH-buffered resistance mechanisms. In orthotopic breast cancer metastasis models, this therapeutic synergy achieved an 82% reduction in pulmonary metastatic burden versus PBS controls, concurrently demonstrating enhanced oxidative stress potentiation and cuproptosis biomarker activation. Wu et al.^93^ designed a biomimetic Cu-ES-polyphenol network integrated with catalase to alleviate the hypoxic TME in osteosarcoma and induce cuproptosis, effectively suppressing tumor growth. Given the propensity of osteosarcoma for early pulmonary metastasis that severely compromises patient prognosis, this strategy further targets metastatic lesions by improving hypoxia within these niches through enhanced oxidative stress, thereby inhibiting pulmonary metastasis progression.

### 3.4 Other synergistic therapy

Beyond the aforementioned synergistic strategies, researchers have explored additional synergistic approaches that integrate distinct therapeutic modalities with cuproptosis to enhance antitumor efficacy and overcome treatment resistance. These include metabolic modulation, energy-based physical therapies, and targeted strategies for challenging metastatic sites such as the brain.

#### 3.4.1 Metabolic modulation

It has been demonstrated that metastatic tumors often exhibit metabolic plasticity and consequently enhance immune escape and treatment resistance^94^^,^^95^. Combined metabolic modulation of cuproptosis death offers a promising strategy for the treatment of cancer metastasis. To intelligently regulate the “OFF-to-ON” catalytic activity of glucose oxidase (GOx, a natural enzyme) while counteracting Cu imbalance-driven metastasis, an acid-responsive GOx-based nanocarrier was constructed through co-assembly of Cu ions and omeprazole (OPZ) on GOx, exposing thiol groups and hydrophobic pockets. Wang et al.^96^ reported that this nanoparticle suppresses pivotal signaling cascades in epithelial–mesenchymal transition and extracellular matrix remodeling to inhibit metastasis, while concurrently intensifying metabolic stress-driven cuproptosis to reverse the immunosuppressive microenvironment. Also regulating glucose metabolism, Lu et al.^97^ constructed a hydrogel system for peritumoral sustained sequential release of dichloroacetate and TPP-CuET. Dichloroacetate was able to shift tumor metabolism away from glycolysis and toward oxidative phosphorylation, which in turn increased the susceptibility of tumor cells to cuproptosis. In an *in vivo* model, this hydrogel system significantly inhibited lung metastasis and lymph node metastasis in triple-negative breast cancer by combining glycolytic modulation and cuproptosis-induced immune activation. Li et al.^98^, on the other hand, focused on the fatty acid metabolic reprogramming process that occurs during lymph node metastasis of oral cancer. They developed a Cu-MOF loaded with orlistat called ORL@Cu-MOF, which releases ORL and Cu ions in response to high GSH in the TME. ORL is able to target and inhibit fatty acid synthase in tumor cells, which not only inhibits lipid uptake and lipid droplet formation in oral cancer cells, but also inhibits lipid metabolic reprogramming, but also promotes the production of ROS and the onset of cuproptosis. In a mouse popliteal lymph node metastasis model, ORL@Cu-MOF was shown to exert significant anti-tumor effects and immune activation in primary tumors and lymph node metastasis.

#### 3.4.2 Combined with sonodynamic therapy (SDT)

**Figure 5:**
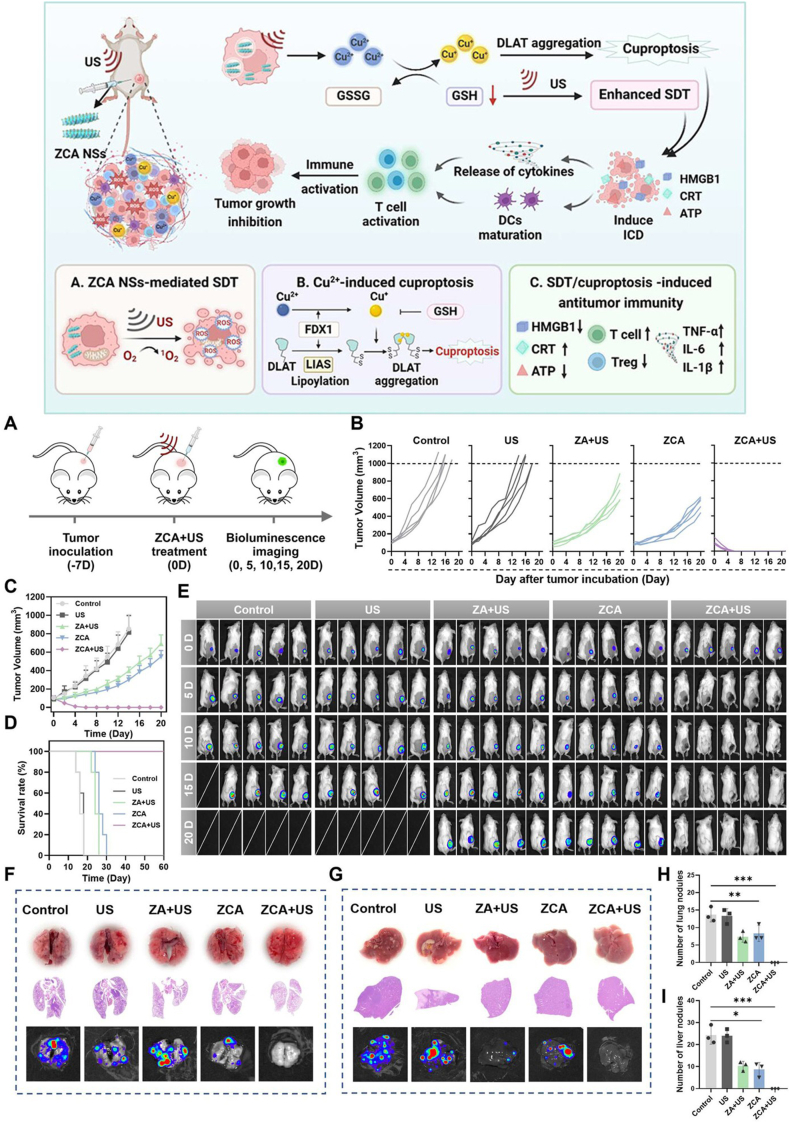
The upper panel shows a schematic diagram of the mechanism of Cu-substituted ZnAl ternary LDH nanosheets (ZCA NSs) combined with SDT for tumor treatment. The lower panel shows: (A) Treatment timeline for breast cancer mice. (B,C) Individual tumor volumes (B) and mean tumor volumes (C) in breast cancer mice treated with control, ultrasound (30 kHz, 10 min), ZA (5 mg/kg, intratumoral injection) + ultrasound, ZCA, or ZCA + ultrasound. (D) Survival rate of tumor-bearing mice over 60 days following different treatments. (E) Bioluminescence imaging of individual mice at days 0, 5, 10, 15, and 20 after various treatments. (F–I) Representative photographs of lung (F) and liver (G) tissues, H&E staining, and bioluminescence imaging of mice after different treatments on Day 30, and the number of metastatic lung nodules (H) and liver nodules (I) in different groups. The results showed that lung metastasis and liver metastasis were significantly less in the ZCA NSs combined with ultrasound group of mice than in the control group. Reprinted with the permission from Ref. 99. Copyright © 2023 American Chemical Society.

Tang et al. developed Cu-substituted ZnAl ternary layered double hydroxide nanosheets (ZCA NSs), where Cu-induced Jahn-Teller distortion optimizes the electronic structure to enhance SDT efficacy. The engineered ZCA NSs amplify oxidative stress by depleting endogenous GSH, thereby potentiating SDT performance and modulating the TME. Furthermore, intracellular Cu overload triggers SDT-amplified cuproptosis, inducing irreversible proteotoxicity. The ZCA NSs-mediated SDT/cuproptosis combination achieved complete eradication of solid tumors *in vivo* while abrogating immunosuppressive TME and suppressing pulmonary/hepatic metastasis (Fig. 5)^99^.

#### 3.4.3 Blood–brain barrier (BBB)-penetrating

To address brain metastasis in small cell lung cancer, Zhang et al.^100^ engineered a syphilis-mimetic TP0751 peptide-decorated, stem cell membrane-coated Cu-MOF (TP-M-Cu-MOF/*siATP7a*) nanodelivery system. This platform demonstrated excellent biocompatibility, efficient transcytosis across the BBB, and specific tumor cell uptake in brain lesions. Mechanistically, it inhibits Cu transport by silencing ATPase copper transporting *α* (*via* encapsulated *siATP7A*), induces intracellular Cu accumulation to trigger cuproptosis, and significantly enhances therapeutic efficacy in small cell lung cancer brain metastasis-bearing murine models.

Table 3 presents cuproptosis-based nanoplatforms relevant to metastasis, focusing on those with validated or inferred anti-metastatic efficacy based on design features and *in vivo* models. From these case studies, we summarize the following several design principles regarding cuproptosis-based nanomedicines against cancer metastasis.

**Table 3:** Cuproptosis-based nanomedicine in anti-metastasis treatment.

Name	Cancer type	Forms of Cu	Treatment method	Drug delivery strategy	Features	Anti-metastatic evidence^a^	Ref.
CuET@PHF	Triple-negative breast cancer	CuET	PTT	Folic acid and sulfhydryl-modified HES; ROS-responsive	Elimination of CSCs and hypoxia	Demonstrated	6
Cuproptosis nanocapsule	Triple-negative breast cancer	PWCu	Radiotherapy	X-ray-responsive	Metastasis-targeted immune-radiotherapy	Demonstrated	7
PTC	Lung cancer	Cu_2_O	PDT	Platelet vesicle	Significant inhibition of metastasis and recurrence; an increase of central memory T cells	Demonstrated	84
*E. coli*@Cu_2_O	Colon cancer	Cu_2_O	Immunotherapy, PTT, ferroptosis	*E. coli* carrier; H_2_S-responsive	PTT-enhanced ICD and ferroptosis	Demonstrated	85
CuS-PEI-siRNA-SFNs	Triple-negative breast cancer	CuS	PTT, CDT, immune activation	Silk fibroin; *PD-L1* siRNA	Reduction of pulmonary metastasis	Demonstrated	86
CSG hydrogel	Colorectal cancer	ES-Cu	Immunotherapy and radiotherapy	Ca^2+^-triggered hydrogel degradation	Downregulating expression of PD-L1; slow and consistent drug release; improving sensitivity to radiotherapy	Demonstrated	87
PCD@CM	Breast cancer	Cu^2+^	Immunotherapy, PTT, and chemotherapy	Responsive to GSH; tumor cell membrane targeting	PTT-enhanced ICD effects; NIR-II window improved light penetration depth	Demonstrated	88
CuP/Er NCP	Breast and colorectal cancer	Cu^2+^	Immunotherapy and ferroptosis	Hybridized core–shell	Dual RCDs and inhibition of metastasis	Implied	89
OMP	Melanoma	Cu-MOF	Immunotherapy	OPDEA, pH-responsive	Aerosol inhalation to reduce pulmonary metastasis	Demonstrated	90
Cel–Cu NP	Triple-negative breast cancer	Cel–Cu	Amplification of oxidative stress	DSPE-PEG_2000_	Inhibition of the NF-*κ*B pathway	Demonstrated	92
Biomimetic copper-based nanocomplex	Osteosarcoma	ES-Cu	Amplification of oxidative stress	cRGD peptid; macrophage-derived membranes	Reduction of pulmonary metastasis	Demonstrated	93
Cu/OPZ@GOx	Triple-negative breast cancer	Cu^2+^	Regulation of glucose metabolism	pH-responsive	Suppression of EMT and ECM remodeling	Demonstrated	96
PED@tCu hydrogel	Triple-negative breast cancer	CuET	Regulation of glucose metabolism and immunotherapy	pH-responsive	Improvement of tumor immune memory and reduction of glycolysis	Demonstrated	97
ORL@Cu-MOF	Oral cancer	Cu-MOF	Immunotherapy	GSH-responsive	Inhibition of lipid metabolic reprogramming	Demonstrated	98
ZCA NSs	Breast and colorectal cancer	Cu-substituted ZnAl nanosheets	SDT	EPR effect	Inhibition of lung and liver metastasis	Demonstrated	99
TP-M-Cu-MOF/*siATP7*a	Small cell lung cancer	Cu-MOF	CDT	TP0751 peptide-modified stem cell membrane	Blocking Cu transport, increasing Cu uptake, and inhibiting brain metastasis	Demonstrated	100

CSCs, cancer stem cells; CSG, Galactose-Alginate; ECM, extracellular matrix; EMT, epithelial–mesenchymal transition; EPR, enhanced permeability and retention; Er, erastin; ES, elesclomol; GSH, glutathione; GOx, glucose oxidase; HES, hydroxyethyl starch; ICD, immunogenic cell death; MOF, metal organic framework; NCP, nanoscale coordination polymers; NP, nanoparticle; OPDEA, polyzwitterion, poly (2-(*N*-oxide-*N*, *N*-diethylamino)ethyl methacrylate); OPz, omeprazole; PD-L1, programmed cell death 1 ligand 1; PEI, poly (ethylene imine); PTT, photothermal therapy; PWCu, polyoxometalate Na_10_[(PW_9_O_34_)_2_Cu_4_(H_2_O)_2_]·18H_2_O; RCDs, regulatory cell death; RGD, Arg-Gly-Asp; SFNs, silk fibroin nanoplatform; ZCA NSs, ZnAl ternary layered double hydroxide nanosheets.

a “**Demonstrated**” means that the entry was validated against metastasis in *in vivo* models (lung metastasis, brain metastasis, distal tumors, etc.), and “**Implied**” means that the anti-metastatic efficacy of the entry was suggested by a design feature or a combination of strategies that were not always directly validated in a metastasis model.

Firstly, selective targeting of metastatic precursors such as CTCs and CSCs is usually accomplished by ligands or antibodies against markers such as CD44 or CD133, with the goal of intercepting metastasis at an early stage^101^^,^^102^. Another recurring strategy is the modulation of the metastatic TME, which is typically characterized by hypoxia, elevated GSH levels, and immune suppression^103^. Nanocarriers responsive to these hallmarks—through pH-sensitive release, ROS amplification, or GSH depletion—allow spatially controlled Cu ion delivery and improve therapeutic selectivity^98^^,^^104^^,^^105^. Additionally, overcoming physiological barriers associated with organ-specific metastasis (*e.g*., the BBB in brain metastasis, lymphatic vessel in lymph node metastasis) has spurred the adoption of surface-engineered NPs capable of targeted homing, using moieties like transferrin, peptides, or exosomal membranes^100^^,^^106^. Furthermore, the co-delivery of cuproptosis-inducing agents with chemotherapeutics, immunomodulators, or phototherapeutic agents within a single nanoplatform reflects a trend toward synergistic multimodal therapy^4^. Importantly, metabolic adaptation represents a pivotal mechanism enabling metastatic survival and colonization, especially under nutrient-deprived or oxidative stress-rich conditions in distant tissues^107^. Therefore, there is great potential to rationally integrate drugs that disrupt metabolic reprogramming, such as inhibition of glycolysis or mitochondrial dysfunction, into cuproptosis nanoplatforms to further inhibit metastatic growth by exploiting metabolic weaknesses specific to diffuse cancer cells^108^. Lastly, ensuring biocompatibility through the use of degradable carriers and stimulus-triggered Cu release systems has become essential for mitigating systemic toxicity.

Most current designs still rely heavily on empirical material selection without fully elucidating the pharmacokinetic and organic effects of Cu-based drugs in the metastatic setting. Furthermore, the exact contribution of cuproptosis to antitumor efficacy relative to other co-administration modalities remains underexplored, raising questions about the mechanistic attribution of the observed results. Studies on how these nanoplatforms interact with evolving metastatic niches are also limited, especially in immune checkpoint-blocked or highly fibrotic microenvironments. Future efforts should aim to rationally integrate cuproptosis into a metastasis-specific therapeutic framework, supported by deeper biological insights and predictive *in vivo* modeling.

## 4 Conclusions and prospects

The association between cuproptosis and cancer is gaining recognition, and changes in CRGs, such as *FDX1*, are being extensively studied in tumors. Cuproptosis presents new therapeutic options for cancer treatment. DDSs based on cuproptosis are drawing interest due to their ability to reduce systemic toxicity and enhance drug efficacy. Additionally, the combination of cuproptosis with other therapies has significantly improved treatment outcomes. Tsvetkov revealed the potential mechanism of cuproptosis independent of other cell death^8^, but a series of unknown issues in the field of cuproptosis still need further research and resolution, such as the specific role and recognition process of Cu ionophores and FDX1 that promote cuproptosis, which deserve further exploration, moreover, the exact mechanism by which abnormal oligomerization of acylated proteins leads to protein toxicity stress and cell death, as well as the morphological characteristics of cells that undergo cuproptosis, are still unknown. Compared to cuproptosis, existing research on ferroptosis is more extensive and comprehensive, and the perspective of ferroptosis can be used to preview potential discoveries in the field of cuproptosis in the future^44^. Both Fe and Cu preparations alone can cause systemic toxicity, and DDS is a good solution to this problem. The sensitivity of various cellular types exhibits variability, necessitating further investigation into the regulatory mechanisms, including the LA pathway and PDH complex, as well as individualized differences encompassing the levels of acylated proteins or GSH content, and mitochondrial respiratory levels within cells. GSH plays an important role in the regulation of ferroptosis, determining the activity of the key enzyme GPX4. Meanwhile, in cell apoptosis, reducing GSH inhibits the process of oxidative stress and cell apoptosis. In cuproptosis, the mechanism of GSH inhibiting cuproptosis by chelating Cu^+^ and inhibiting acylation may be related to the mechanism of cell apoptosis. Furthermore, similar to the considerable potential of ferroptosis in causing ischemic organ damage and degenerative diseases beyond cancer, the diagnostic and therapeutic importance of cuproptosis in inflammatory bowel disease, osteoporosis, and cardiovascular disease warrants significant consideration^109^.

**Figure 6:**
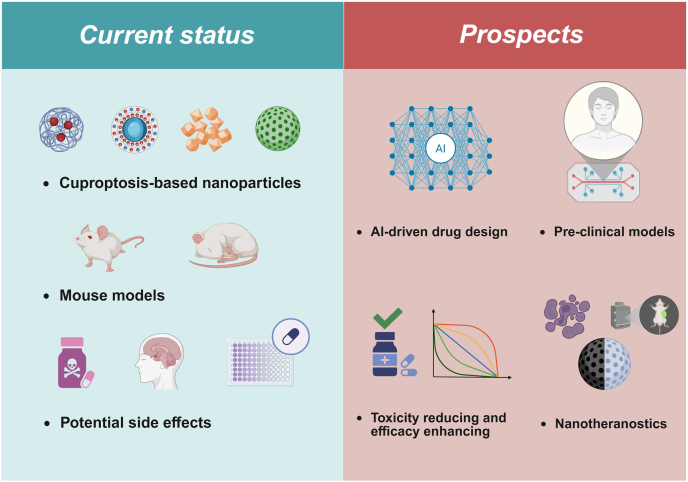
Current status and prospects of cuproptosis-based nanomedicine. Although several studies have developed cuproptosis-based nanomedicine for treating cancer metastasis, this strategy continues to face significant challenges. AI-driven drug design may enhance targeting precision and delivery efficiency to achieve toxicity reduction and efficacy enhancement. Concurrently, advances in preclinical models provide foundations for more clinically translatable drug development. The innovation of nanotheranostic platforms could enable multifunctional capabilities within single formulations, contributing to improved clinical management for patients with metastatic cancer. AI, artificial intelligence. Created with BioRender.com.

Cuproptosis-based DDSs are set to revolutionize cancer treatment, offering targeted, effective therapies that integrate various forms of RCDs with other therapeutic modalities. Despite demonstrating significant therapeutic potential in current research, cuproptosis-based nanomedicine strategies face substantial obstacles prior to clinical translation (Fig. 6). First, the molecular mechanisms of cuproptosis require further elucidation; deeper mechanistic insights could reveal additional therapeutic targets or pathways, enabling targeted therapies that surpass current treatment efficacy. Multi-omics technologies provide foundational insights through spatiotemporal multidimensional characterization of tumor tissues and metastasis. Furthermore, preclinical modeling limitations impede clinical translation—most studies rely on murine models despite substantial biological disparities between mice and humans that create translational chasms. Additionally, cuproptosis-based nanomedicine must address biosafety challenges. Although Cu serves as an essential trace element widely present in human tissues, the cytotoxicity of excessive Cu ions necessitates careful consideration, particularly for Cu-based nanomaterials, including metal organic frameworks or nanospheres^40^. Passive accumulation *via* the EPR effect directs significant nanodrug deposition in hepatic and renal tissues during excretion, mandating thorough investigation of potential hepatorenal toxicity^110^. Separately, while certain ES-based nanotherapeutics contain no Cu ions, ES itself exhibits reported neurotoxicity concerns. These collective biosafety issues constitute major barriers to clinical translation. Modifications through structural refinement or optimized delivery modalities may mitigate these concerns^111^. Specifically, targeted delivery systems with controlled sustained release kinetics could simultaneously enhance therapeutic outcomes while reducing off-target effects and biological toxicity^112^.

Current research predominantly focuses on cuproptosis-based nanomedicine strategies for pulmonary metastasis, yet metastasis to other organs (*e.g*., hepatic and cerebral) and lymph node dissemination hold distinct clinical significance in specific malignancies. While brain metastasis necessitates specialized nanocarrier designs to traverse the BBB for enhanced drug deposition and therapeutic efficacy, lymph node metastasis remains a critical prognostic determinant in cancers such as oral squamous cell carcinoma^113^. Our prior review systematically analyzed nanoparticle-based lymph node targeting strategies and their physicochemical determinants, providing foundational insights for developing cuproptosis-optimized nanoplatforms against lymphatic dissemination^106^^,^^114^^,^^115^. In recent work, we engineered a cuproptosis-inducing nanotherapeutic platform that effectively suppresses oral squamous cell carcinoma lymph node metastasis, with synergistic *α*PD-1 immunotherapy demonstrating enhanced therapeutic efficacy^98^.

**Figure 7:**
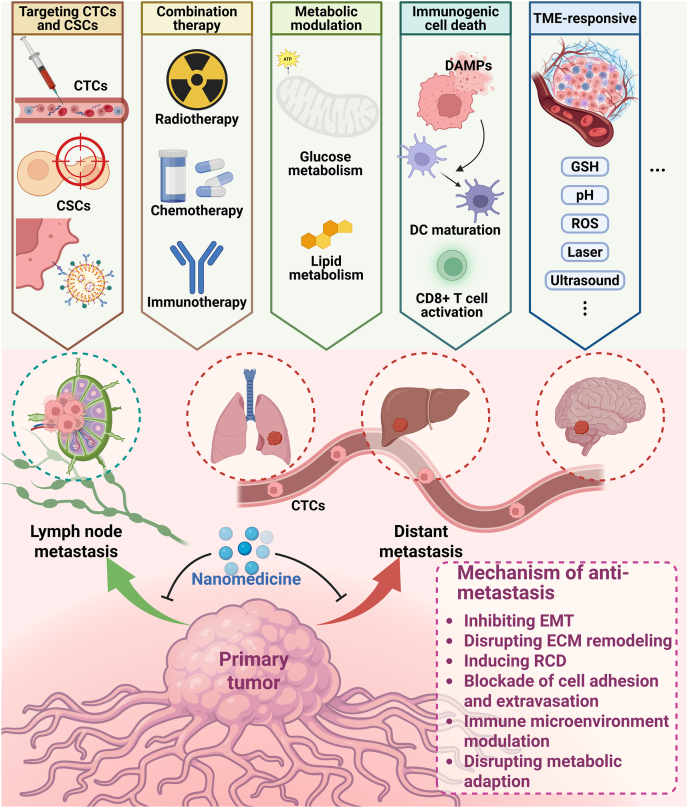
Design and mechanism of cuproptosis-based nanomedicine in anti-metastasis treatment. Anti-metastatic nanomedicines are designed to precisely target metastatic precursors (circulating tumor cells and cancer stem cells (CSCs)), remodel the immunosuppressive microenvironment, and disrupt key steps in the metastatic cascade, such as epithelial–mesenchymal transition (EMT), extracellular matrix (ECM) remodeling, and metabolic adaptation. By incorporating stimuli-responsive systems and multifunctional components, these nanoplatforms not only enhance selectivity and efficacy but also offer synergistic therapeutic potential through combination with immunotherapy or programmed cell death inducers. Created with BioRender.com.

**Figure 8:**
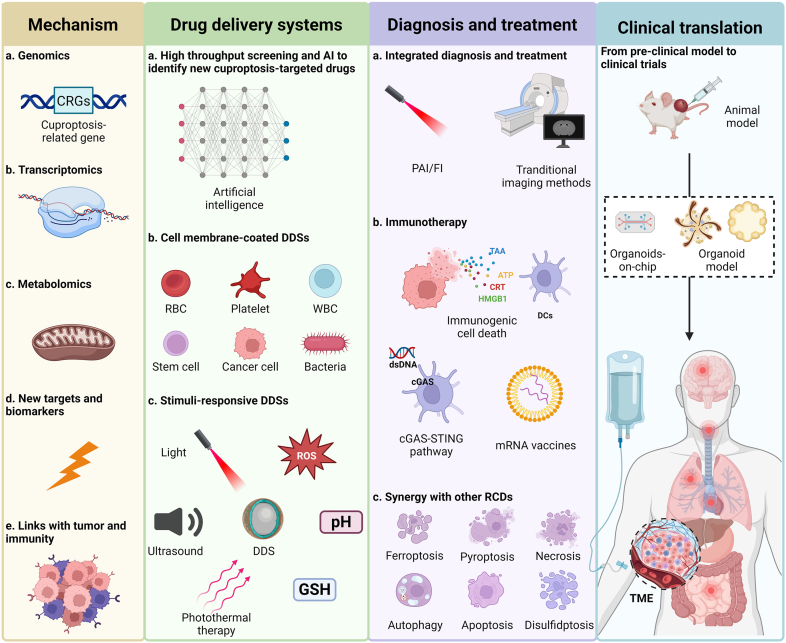
Future prospects of cuproptosis-based DDSs for tumor treatment. The mechanism of cuproptosis needs to be further elucidated by multi-omics analyses to obtain more information about the targets and biomarkers of cuproptosis. In addition, technologies such as artificial intelligence (AI) and high-throughput screening can be utilized to find more effective inducers of cuproptosis. As for DDSs, engineered cell membrane-coated DDSs exhibit great potential in biocompatibility and tumor targeting. And multi-stimuli-responsive property is of benefit for improving the selectivity of drug delivery. Cuproptosis-based DDSs can function as good tools for cancer treatment and diagnosis, like integration of diagnosis and treatment, immunotherapy, and synergized therapy with other RCDs. However, most DDSs are facing the challenge of clinical translation, and the organoid model is promising for bridging the gap. Created with BioRender.com.

Optimizing the design of cuproptosis-based nanomedicine strategies requires rational selection of delivery methods and targeting approaches. Metastatic lesions present distinct challenges compared to primary tumors, where effective drug delivery must overcome pharmacokinetic barriers, enhanced permeability and retention effect limitations, and critical factors such as lymphatic system penetration and accumulation that significantly influence nanocarrier performance. Surface modifications, including specialized coatings enhance selectivity for inducing tumor-specific Cu deposition, thereby improving therapeutic efficacy. Nanocarriers exhibiting high payload capacity, tunable functionality, and pH-responsive degradation represent ideal vehicles for Cu ion or Cu ionophore delivery (Fig. 7). Beyond therapeutic applications, cuproptosis offers diagnostic value through cuproptosis-related gene signatures that enable tumor progression monitoring and prognosis prediction. Cuproptosis-based nanotheranostic platforms integrated with advanced imaging techniques achieve precise tumor visualization, while artificial intelligence implementation enhances diagnostic accuracy and efficiency^116^. Furthermore, AI-driven high-throughput screening of naturally benign compounds provides promising alternatives to advance precision medicine by minimizing systemic toxicity while enhancing drug efficacy, warranting expanded preclinical and clinical validation (Fig. 8). Collectively, cuproptosis-driven nanomedicine is emerging as a pivotal modality for metastatic cancer therapy. Its combinatorial potential with conventional therapies (chemotherapy, immunotherapy) and emerging modalities (SDT, PTT) reveals transformative prospects^117^. We advocate for intensified translational efforts to accelerate clinical translation of these innovations, ultimately advancing survival outcomes for patients with metastatic malignancies^111^.

## Author contributions

Kan Zhou: Investigation, Methodology, Conceptualization, Writing-Original Draft, Visualization, Writing–Review & Editing. Zi-Zhan Li: Investigation, Methodology, Conceptualization, Writing-Original Draft, Writing–Review & Editing. Yi Liu: Investigation, Methodology, Conceptualization, Writing-Original Draft, Writing–Review & Editing. Lei-Ming Cao: Methodology, Conceptualization, Writing-Original Draft, Writing–Review & Editing. Han-Yue Luo: Methodology, Conceptualization, Writing-Original Draft, Writing–Review & Editing. Guang-Rui Wang: Methodology, Conceptualization, Writing-Original Draft, Writing–Review & Editing. Kang-Ning Wang: Methodology, Conceptualization, Writing-Original Draft, Writing–Review & Editing. Jinmei Wu: Methodology, Conceptualization, Writing-Original Draft, Writing–Review & Editing. Bing Liu: Supervision, Writing-Review & Editing, Project Administration. Zhiyong Song: Supervision, Writing-Review & Editing, Project Administration. Lin-Lin Bu: Conceptualization, Supervision, Project Administration, Funding Acquisition, Writing-Review & Editing. All authors have reviewed and approved the final version of this manuscript for publication. Each author agrees to be accountable for all aspects of the work in ensuring that questions related to the accuracy or integrity of any part of the work are appropriately investigated and resolved.

## Conflicts of interest

The authors declare no conflicts of interest.
